# Assessing the contribution of myelofibrosis to a leukoerythroblastic blood picture

**DOI:** 10.1016/j.htct.2025.103735

**Published:** 2025-02-07

**Authors:** Stephen E. Langabeer

**Affiliations:** Cancer Molecular Diagnostics, St. James's Hospital, Dublin, Ireland

Dear editor,

A leukoerythroblastic blood picture (LBP) is one in which myeloid precursors (myelocytes or promyelocytes) and nucleated red blood cells are present in the peripheral blood on morphological examination. Other features are variable and include poikilocytosis, a low-grade reticulocytosis and a normocytic normochromic anemia. The underlying causes of an LBP are many and include bone marrow-infiltrating solid tumors, primary hematological malignancies, haemolytic diseases, liver diseases, megaloblastic anemia, hemorrhage and acute infection. Its presence in patients with malignancies can be an indicator of disease progression associated with an adverse prognosis.^1^ Of the hematological malignancies, an LBP can be a frequent but not invariable presenting feature of the myeloproliferative neoplasms (MPN) of primary myelofibrosis (PMF), post-polycythemia vera myelofibrosis (post-PV MF) and post-essential thrombocythemia myelofibrosis (post-ET MF) and as such remains a minor diagnostic criterion in current classification systems of these malignancies.^2^ PMF is driven by acquired mutations of *JAK2, CALR* exon 9 or *MPL* exon 10 with further somatic mutations contributing to disease phenotype and prognosis.^2^ Given the association of an LBP with a diagnosis of PMF/post-PV MF/post-ET MF, up-front screening for driver mutations in patients with an LBP requires evaluation in the context of the appropriate deployment of molecular diagnostics.

A review of requests was performed for MPN molecular diagnostics from January 2006 to June 2023 inclusive at a center for molecular diagnostics of hematological malignancies that performs >2000 diagnostic MPN panels per annum. A total of 206 requests were identified with an LBP included within the clinical details on the request form. Of these 206, 94 had additional clinical details suggestive of PMF or post-PV/post-ET MF of either one or more of the following: constitutional symptoms, splenomegaly and/or hepatomegaly, cachexia, anemia, pruritus, raised serum lactate dehydrogenase, or tear drop poikilocytes present on the peripheral blood film. Testing was performed on DNA extracted from peripheral blood (*n* = 198) or bone marrow aspirate (*n* = 8) samples. Patient consent for MPN mutation testing was required at the referring center. Overall, PMF-associated driver mutations were detected in 64 (31.1 %) patients with an LBP. Driver mutations were detected in 16 patients in the LBP only group (14.3 % - *JAK2* V617F: *n* = 12; *CALR: n* = 2; and *MPL: n* = 2) and in 48 patients with LBP and an additional feature(s) (51.1 % - *JAK2* V617F: *n* = 33; *CALR: n* = 10; and *MPL: n* = 5).

Potential confounding factors in this brief review include the lack of or incomplete clinical information provided on laboratory request forms: a common problem in molecular pathology, and the potential non-recognition of ‘triple-negative’ PMF patients.^3^^,^^4^ A possible bias skewing in favor of molecular detection is that the majority of referrals were from hematologists, likely to be alert to any underlying diagnosis. Despite these limitations, this review highlights the relatively high incidence of myelofibrosis-associated driver mutations in patients with an LBP which consequently is an important indication for performing bone marrow aspiration and biopsy.^5^ Furthermore, the high incidence of mutations in patients with additional hematological or clinical features associated with PMF/post-PV-MF/post-ET MF re-enforces the role of LBP as a minor diagnostic criterion for these malignancies.

## Funding

This research received no specific grant from any funding agency in the public, commercial, or not-for-profit sectors

## Conflicts of interest

The author declares no potential conflicts of interest with respect to the research, authorship, and/or publication of this article.
